# 

**Published:** 1943

**Authors:** 


### CONTENTS.

| | Page |
|---|---|
| The Future of Health and Hospital Services from an Architect's Point of View. By Charles E. Elcock, F.R.I.B.A. .. .. | 1 |
| Social and Economic Conditions and the Incidence of Rheumatic Heart Disease. By G. H. Daniel, D.Phil. .. .. .. | 13 |
| Reviews of Books .. .. .. .. .. .. .. .. | 27 |
| Editorial Notes .. .. .. .. .. .. .. .. | 32 |
| Obituaries: Emeritus Professor F. H. Edgeworth, M.A., M.D. (Cantab.), D.Sc. (Lond.) .. .. .. .. | 33 |
| Ronald Henry Tasker, M.R.C.S., L.R.C.P. .. .. | 35 |
| John Lacy Firth, M.D., M.S.(Lond.), F.R.C.S. .. | 36 |
| The Medical Library of the University of Bristol .. .. .. | 37 |
| Publications Received .. .. .. .. .. .. .. | 40 |
| Local Medical Notes .. .. .. .. .. .. .. | 41 |

